# Deciphering Scorpion Toxin-Induced Pain: Molecular Mechanisms and Ion Channel Dynamics

**DOI:** 10.7150/ijbs.109713

**Published:** 2025-04-21

**Authors:** Dangui He, Yining Lei, Haixin Qin, Zhijian Cao, Hang Fai Kwok

**Affiliations:** 1 Department of Biomedical Sciences, Faculty of Health Sciences, University of Macau, Avenida de Universidade, Taipa, Macau SAR.; 2 National "111" Center for Cellular Regulation and Molecular Pharmaceutics, Key Laboratory of Fermentation Engineering (Ministry of Education), Hubei University of Technology, Wuhan 430068, Hubei Province, China.; 3 Center for Evolution and Conservation Biology, Southern Marine Science and Engineering Guangdong Laboratory (Guangzhou), Guangzhou 511458, Guangdong Province, China.; 4 Institute of Translational Medicine, Faculty of Health Sciences, University of Macau, Avenida de Universidade, Taipa, Macau SAR.

**Keywords:** scorpion toxin, pain, ion channel, molecular mechanism, analgesic therapy

## Abstract

Scorpion toxins significantly disrupt the normal function of ion channels, leading to abnormal nerve excitability and severe pain responses. Notably, α-type sodium channel toxins (α-NaTx) and β-type sodium channel toxins (β-NaTx) target sodium channels through distinct mechanisms: α-NaTx prolongs channel opening, while β-NaTx lowers the activation threshold, resulting in persistent nerve overexcitation and heightened pain. This review synthesizes current knowledge on pain-inducing venom peptides isolated from various scorpion species, elucidating the underlying molecular mechanisms involving ion channels. Furthermore, it explores the potential applications of these toxins in scientific research and drug development, highlighting their significance in advancing our understanding of pain mechanisms and facilitating the development of novel analgesic therapies.

## 1. Introduction

As early as the Silurian Period, about 435 million years ago, the ancestors of scorpions—Eurypterida—began adapting from an aquatic environment to life on land, much like other arthropods 1. Since then, the basic morphology and way of life of scorpions have remained largely unchanged, earning them the title of 'living fossil'. Scorpions rely on venom as a critical survival tool. They possess a stinger used to inject venom into preys or predators at the tip of their tail. Scorpion venom is a complex mixture of bioactive substances, including neurotoxins, enzymes, antimicrobial peptides, and so on 2, 3. The neurotoxins disrupt nerve conduction in preys by interfering with ion channels, such as sodium, potassium, calcium, or chloride causing paralysis or death 4. While most scorpion venoms are harmless to humans, a few species can produce venom potent enough to pose serious threats. In Mexico, 800 to 1,000 people die each year due to scorpion stings 5. There are significant differences in the structure and function of the venom components of different scorpion species, and these differences are related to their ecological environments, prey types, and hunting methods.

The historical connection between scorpion venom and pain dates back to ancient times. When faced with scorpion stings, early human civilizations quickly realized that their venom could cause severe pain 6. Although medical knowledge at the time was limited, these early documents laid the groundwork for modern scientific research into scorpion venom. It was observed that scorpion stings not only caused excruciating pain but could also lead to paralysis, muscle spasms, and even severe neurological symptoms, particularly in encounters with highly venomous species. These extreme pain reactions drew the attention of modern scientists to the components of scorpion venom. Among these components, peptide toxins are identified as the primary agents responsible for pain. For example, there are many pain-causing toxin peptides in *Buthus martensii* Karsch, such as BmKI, MkTxIII. These toxins can disrupt the normal activity of neurons, with pain being the result of abnormal stimulation or activation. Studies have shown that scorpion toxins can interfere with the normal function of ion channels, particularly sodium, potassium, and calcium channels in the nervous system, leading to the sensation of pain.

Recent research on scorpion toxins has advanced significantly, focusing on their molecular diversity, structural characteristics, ion channel interactions, and therapeutic applications. Modern studies have identified various types of toxins in scorpion venom, particularly those that target ion channels, including specific modulators of sodium, potassium, and calcium channels 3, 7-14. Researchers have analyzed the gene structures and protein sequences of many scorpion toxins using transcriptomic and proteomic technologies, revealing a remarkable degree of molecular diversity. This diversity is evident not only in the sequence variations of the toxins but also in their structural folding patterns and the specific binding sites they target on different ion channels. The pharmacological potential of scorpion toxins has also gained widespread recognition, particularly in areas such as pain management, anticancer therapies, and immune modulation. Recent research has demonstrated that certain scorpion toxins possess significant analgesic properties 15-17. Interestingly, scorpion venoms also contain analgesic peptides. For instance, BmK AGAP, a 66-amino acid peptide isolated from *Buthus martensii* Karsch, exerts potent analgesic effects by inhibiting mitogen-activated protein kinase (MAPK) signaling pathways in both peripheral nociceptors and spinal cord neurons 18. This suppression of MAPK activity reduces pro-inflammatory cytokine release and attenuates central sensitization, thereby alleviating inflammatory and neuropathic pain. This dual role of scorpion toxins—inducing pain in prey while offering therapeutic potential in pain management—highlights their complex interplay with ion channels and underscores their value in biomedical research. Beyond their pain-relieving effects, scorpion toxins have shown anti-tumor activity, with some toxins inducing apoptosis in tumor cells or inhibiting their proliferation. This positions scorpion toxins as promising candidate molecules for developing novel anticancer therapies.

Although some research progresses have been made in understanding the mechanisms by which scorpion toxins induce pain via ion channels, the underlying interactions remain complex and are not yet fully elucidated. Research has shown that scorpion toxins regulate the activation and inactivation of ion channels, such as sodium, potassium, and calcium, by binding to specific sites, leading to excessive neuronal excitation and abnormal pain signal transmission 10, 19-22. However, the precise modes of action, binding sites, and structure-function relationships between toxins and various ion channels require further systematic investigation and review. In particular, the interactions between different toxins across multiple ion channels and their combined effects on pain mechanisms are not yet fully understood, and current research lacks a unified theoretical framework. Therefore, more comprehensive studies and reviews are necessary to uncover the molecular mechanisms underlying the interaction between scorpion toxins and ion channels in promoting pain, ultimately providing clearer pathways for the development of novel analgesics.

## 2. Scorpion venom peptides acting on ion channels

Scorpion venom consists of a complex mixture of biologically active compounds, primarily proteins and peptides, which vary across different species 23, 24. The major components include neurotoxins, enzymes, antimicrobial peptides, and other active substances. Neurotoxins are particularly important, targeting the nervous system of preys or predators by disrupting nerve signal transmission 25, 26. These toxins mainly affect ion channels, such as sodium, potassium, calcium, and chloride. Notably, α-type sodium channel toxins (α-NaTx) extend the opening time of sodium channels, while β-type sodium channel toxins (β-NaTx) lower the activation threshold, both leading to excessive neural activity and pain. In addition to neurotoxins, scorpion venom contains enzymes like phospholipases, hyaluronidases, and proteases 13, 27, 28. These enzymes damage cell membranes and tissues, facilitating venom spread and enhancing toxicity. For instance, hyaluronidase degrades the extracellular matrix, promoting venom diffusion in tissues. Scorpion venom also possesses antimicrobial properties, protecting scorpions from infections during predation or when exposed to environmental wounds by inhibiting the growth of bacteria and fungi 29. This highly effective mechanism ensures the success of scorpion predation. In defense, scorpion venom deters predators by causing severe pain and discouraging attacks. While the venom of many scorpion species only causes temporary pain and discomfort in humans and larger animals, some highly toxic species have venom potent enough to pose significant threats and, in extreme cases, can be fatal in extreme cases. Scorpion venom is a specialized evolutionary tool for hunting and self-defense 30. Its diverse bioactive components and complex mechanisms allow scorpions to thrive in various environments. The study of scorpion venom not only reveals the survival mechanisms of this ancient species but also offers promising avenues for innovation in medicine and drug development 13. Ongoing research into the complex composition of scorpion venoms may lead to groundbreaking applications in various biological and medical fields 31, particularly concerning neurotoxins, which are the most thoroughly investigated components. These neurotoxins play a central role in scorpion venoms, primarily targeting neural transmission by affecting ion channels, leading to paralysis, muscle spasms, or even death through the disruption of nerve signal propagation 32-34. These toxins mainly include sodium channel toxins (NaTx), potassium channel toxins (KTx), calcium channel toxins (CaTx), chloride channel toxins (ClTx), and TRP channel toxins (TRPTx), all of which can alter the permeability of ion channels.

### 2.1. NaTx

#### 2.1.1. α-NaTx

Alpha-type sodium channel toxins are one class of neurotoxins predominantly found in scorpion venoms across various scorpion species 35. Typically composed of 60 to 70 amino acids, these toxins possess a complex three-dimensional structure characterized by multiple disulfide bonds, which confers high stability and functional activity in physiological environments 35-37. The primary mechanism of action of α-NaTx involves binding to specific regions of voltage-gated sodium channels, inducing conformational changes that enhance channel activation at lower membrane potentials. This binding facilitates the earlier opening of sodium channels during membrane depolarization, resulting in a rapid influx of sodium ions into the cell 38. Furthermore, α-NaTxs significantly inhibit sodium channel inactivation, allowing channels to remain open longer after activation 10, 31, 39, 40. This prolonged opening leads to neuronal overexcitation and sustained electrical signaling, ultimately triggering intense pain perception. For instance, BmKI 41 features a specific peptide sequence that markedly enhances the activation of the channel Nav1.7, contributing to significant pain responses. Similarly, the α-NaTx Ts1 from *Tityus serrulatus* in Brazil affects sodium channel inactivation, eliciting strong pain responses and notable inflammatory reactions in mouse models 42. Due to their selective effects on sodium channels, α-NaTxs are valuable structural and mechanistic probes for studying sodium channelopathies and guiding the design of subtype-selective sodium channel modulators. Their subtype-specific binding modes provide critical insights into channel gating mechanisms, which can be exploited to develop novel analgesics. Researchers aim to minimize side effects and enhance drug efficacy by focusing on specific sodium channel subtypes. Additionally, these toxins serve as valuable tools in neurophysiological research, facilitating a deeper understanding of the structure-function relationship of sodium channels. As research on these toxins progresses, scientists are uncovering their complex mechanisms of action, paving the way for novel drug design and improved pain management strategies.

#### 2.1.2. β-NaTx

Beta-type sodium channel toxins are another key class of neurotoxins in scorpion venoms, primarily acting on voltage-gated sodium channels 43. Unlike α-NaTxs, which interfere with sodium channel inactivation, β-NaTxs target the activation mechanism of these channels. Typically composed of 50 to 70 amino acids, β-NaTxs have multiple disulfide bonds contributing to their stability and functional activity 44, 45. They mainly interact with the sodium channel's voltage sensor region, altering the sensor's dynamics and enabling earlier activation upon depolarization. By lowering the activation threshold of the sodium channel, β-NaTxs allow sodium ions to enter neurons at lower membrane potentials, resulting in neuronal overexcitation 46, 47. However, unlike α-NaTxs, β-NaTxs have minimal impact on the inactivation process of sodium channels, focusing primarily on the activation phase 47, 48. Physiologically, β-NaTxs make neurons more prone to firing action potentials by lowering the activation threshold of sodium channels, leading to effects such as muscle spasms, pain, and other nervous system reactions. At higher doses, they can cause severe nervous system dysfunction, potentially resulting in central nervous system abnormalities. For example, CssII from the Brazilian yellow scorpion effectively reduces the activation threshold of Nav1.2 channel, enhancing neural signal transmission 9, 49. Due to their selective targeting of specific sodium channel subtypes, they hold potential as targets for the development of analgesic drugs. Research on the structure-function relationship of β-NaTxs with sodium channels continues to reveal crucial insights into neural signal transduction and channel activation. Such study provides a foundation for the future development of neuromodulatory therapies aimed at treating pain and other neurological conditions.

### 2.2. KTx

Potassium ion channel toxins in scorpion venoms are specialized peptide toxins that target potassium channels, critical for maintaining cellular excitability and homeostasis 12. These toxins mainly consist of short peptide chains usually between 20 and 40 amino acids stabilized by pairs of disulfide bonds (belonging to α-KTx, γ-KTx, κ-KTx, ε-KTx or λ-KTx), and also include some long-chain members with more than 50 amino acids hinged by disulfide bridges (belonging to β-KTx or δ-KTx) 50. Such structural feature confers high stability and allows precise interaction with specific potassium channels 51-53. As a result, KTxs can exert potent effects even at low concentrations. By selectively binding to potassium channels, KTxs disrupt the normal flow of potassium ions across the cell membrane, producing various physiological effects, especially on the nervous and muscular systems. Potassium channels play a key role in returning depolarized neurons or muscle cells to their resting states after an action potential 51, 54, 55. When KTxs interfere with these channels, they impair cellular repolarization, leading to prolonged depolarization and excessive action potential firing, particularly in neurons and muscles. KTxs primarily target two types of potassium channels: voltage-gated potassium channels (Kv) and calcium-activated potassium channels (KCa). Kv channels are crucial for restoring the resting membrane potential after an action potential 14, 51, 56, 57. Potassium efflux through these channels helps repolarize the membrane, terminating the electrical signal. KCa channels, on the other hand, respond to intracellular calcium levels and help regulate cellular excitability in neurons and muscle cells. When KTxs block these channels, they induce neuronal hyperexcitability by preventing the proper repolarization of action potentials 51, 55. This prolongs depolarization, leading to overactive neuronal firing and then in turn, pain sensations that manifest as hyperalgesia (increased sensitivity to pain). In muscle cells, this blockade can cause muscle dysfunction, including spasms and paralysis, due to the disrupted ion balance. Due to their high specificity for potassium channels, KTxs are invaluable tools in neurobiology and electrophysiology research. They enable scientists to study the roles of potassium channels in critical physiological processes such as nerve conduction, muscle contraction, and cardiac rhythm regulation 54. Additionally, KTxs are used to explore pathophysiological conditions related to potassium channel dysfunction, such as epilepsy, chronic pain, and arrhythmias, making them potential candidates for the development of targeted therapies 14, 53. In summary, KTxs from scorpion venoms are highly potent blockers modulators of potassium channel activity, significantly affecting neuronal excitability and muscle function. Their selective mechanism of action continues to provide valuable insights into the physiological importance of potassium channels and therapeutic opportunities in ion channel-targeting drugs.

### 2.3. CaTx

Calcium toxins from scorpion venoms are a class of toxic peptides that specifically target voltage-gated calcium channels, disrupting various physiological processes by altering the transmembrane flow of calcium ions 58. Calcium channels are crucial for neural signal transmission, muscle contraction, and hormone secretion. Therefore, when calcium toxins interfere with these channels, they trigger a cascade of reactions in both the nervous and muscular systems 59. Typically composed of 20 to 40 amino acids CaTx has a highly stable structure reinforced by multiple disulfide bonds. These toxins exhibit selectivity for different types of calcium channels, including L-type, N-type, and P/Q-type channels, effectively disrupting their normal function 8. CaTxs operate through two primary mechanisms. On the one hand, CaTxs enhances calcium channel activation, increasing calcium ions' influx into cells. On the other hand, they inhibit calcium channel inactivation, prolonging the open state and allowing for a sustained influx of calcium 60, 61. This continuous calcium influx can cause neuronal hyperexcitability, manifesting as symptoms like pain and muscle spasms. Additionally, CaTxs can affect the calcium channels of cardiomyocytes, potentially resulting in cardiovascular complications such as arrhythmias. Physiologically, CaTxs primarily cause overactivation of the nervous and muscular systems. Given the role of calcium channels in neural signaling 61, CaTxs heighten neuronal excitability through excessive calcium entry, leading to intense neural responses. They may also cause prolonged muscle contraction, resulting in spasms and stiffness. When acting on the calcium channels in heart cells, CaTxs can disrupt normal cardiac rhythm, contributing to cardiac arrhythmias. CaTxs hold significant value in scientific research and drug development. Since calcium channels are implicated in conditions such as neuropathic pain, epilepsy, migraines, and cardiovascular diseases, CaTxs provide valuable tools for investigating these pathological mechanisms 7, 60. Their high selectivity allows researchers to probe the role of calcium channels in different disease states, offering new perspectives for the development of calcium channel-targeting drugs. By examining the interaction between CaTxs and calcium channels, scientists can gain deeper insights into the fundamental roles of these channels playing in the nervous and cardiovascular systems, ultimately facilitating the design of novel therapeutic agents. In brief, CaTxs from scorpion venoms disrupt the balance of intracellular calcium ions, triggering a range of physiological reactions by precisely regulating calcium channel activity. This makes them not only essential tools for studying bioelectric activity and calcium channel function but also promising candidates for future drug development.

### 2.4. ClTx

Chlorotoxin is a significant toxin found in scorpion venom, primarily targeting the chloride channel to regulate the transmembrane flow of chloride ions 62. This regulation affects the physiological functions of both nerves and muscles. Chloride channels are crucial for maintaining resting membrane potential, regulating cell excitability, and facilitating nerve signal transmission. Thus, chlorotoxin interference can trigger a range of physiological responses 63. Typically composed of short-chain polypeptides with multiple disulfide bonds, ClTxs remain stable in various environments. By binding to specific sites on chloride channels, they enhance the duration of channel opening, resulting in increased chloride ion influx. This alteration can lead to either depolarization or hyperpolarization of the cell membrane, subsequently affecting neuronal excitability and muscle contractility 64, 65. The physiological effects of ClTxs are primarily reflected in impaired muscle function. They increase the excitability of nerve cells, causing pain and other neurological symptoms. In muscle cells, ClTxs may disrupt normal contraction and relaxation processes, leading to muscle spasms or weakness. Additionally, chloride channels are vital for the electrophysiological stability of cardiac cells, and 'ClTx's effects may result in arrhythmias or other cardiac dysfunctions 66. In scientific research and drug development, ClTxs serve as valuable tools for understanding chloride channel function, particularly in the context of diseases related to chloride channel dysfunction 65, 67. Researchers can develop targeted therapies by investigating the interactions between ClTxs and chloride channels, paving the way for new treatments for related disorders. Briefly, ClTxs show significant research and application value by modulating chloride channel function and inducing a variety of physiological reactions.

### 2.5. TRPTx

Transient receptor potential (TRP) channels are a vital class of ion channels found throughout mammalian sensory neurons, playing key roles in various physiological processes, including the perception of temperature, pain, mechanical stimulation, and chemical stimuli 68, 69. Different TRP channel subtypes, such as TRPV, TRPA, and TRPM, exhibit distinct physiological functions and responses to stimuli, particularly in regulating sensory signal transmission and contributing to pain perception and inflammatory responses. Only a limited number of scorpion toxins that modulate TRP channels have been identified 70-72. WaTx primarily targets the channel TRPA1, which is crucial for sensing high temperatures and mechanical stimuli. It activates TRPA1 by penetrating the plasma membrane and reaching intracellular sites modified by reactive electrophiles 19. This toxin stabilizes TRPA1 in a unique active state, characterized by prolonging channel opening and reducing Ca²⁺ permeability. BmP01, a peptide derived from the venom of the scorpion *Mesobuthus martensii* Karsch (previously named as *Buthus martensii* Karsch), was initially reported as an inhibitor of voltage-gated (Kv) channel subtypes. However, it was later found to induce pain in mice by targeting TRPV1 channel 73. These scorpion toxin peptides not only enhance our understanding of the role of TRP channels in pain and sensory transmission but also hold potential for biomedical applications in pain management and treatment 72, 74. By investigating the interactions between these toxins and TRP channels, researchers can establish a foundation for developing new targeted therapies to alleviate pathological conditions associated with TRP channel dysfunction.

## 3. Scorpion venom peptides with pain-inducing properties

People fear scorpions because their stings can cause redness, swelling, and intense pain, symptoms closely linked to scorpion venom. When a scorpion stings, it injects venom directly into the tissue, where proteins and enzymes stimulate local tissues, activate the immune system, and trigger inflammatory responses. Inflammatory mediators released in the body, such as histamine, lead to inflammation, resulting in redness and swelling. Additionally, certain peptides in scorpion venom can act directly on the nervous system, particularly by affecting sodium ion channels, which disrupt nerve signal conduction and induce severe pain. Pain perception is partly regulated by three VGSC subtypes—Nav1.7, Nav1.8, and Nav1.9—all of which are expressed in nociceptors and play central roles in transmitting pain signals to the central nervous system.

*Tityus serrulatus*, Brazil's most recognized scorpion species, causes a wide range of symptoms in sting victims, including vomiting, abdominal pain, agitation, excessive salivation, and fever. Numerous toxin peptides have been isolated from this species, including Ts1, Ts2, Ts3, and Ts19. Among these, Ts1 is the most abundant and toxic component. Classified as a β-NaTx, Ts1 modulates sodium channel activation, particularly Nav1.6 and Nav1.3, causing these channels to open at the resting potential 42, 75. Experimental intracerebral injection of Ts1 in rats induces symptoms such as hind limb paralysis and severe respiratory distress, ultimately leading to death, highlighting its potent neurotoxicity 76. Another notable peptide, Ts5, is an α-NaTx that delays the inactivation of voltage-dependent sodium channels 77. Although Ts5 represents only about 2% of the venom, it is highly toxic and targets a wide range of sodium channels, including Nav1.2, Nav1.3, Nav1.4, Nav1.5, Nav1.6, and Nav1.7. In contrast, Ts6 interacts with potassium channels, blocking high-conductance calcium-activated potassium channels and exhibiting varying affinities for different potassium channel subtypes 78. This diversity in channel interaction highlights the complexity of *Tityus serrulatus* venom and its evolved mechanisms for prey immobilization and predator deterrence.

*Mesobuthus martensii* Karsch*,* a species widely distributed across East Asia, has a long-standing history in traditional Chinese medicine due to its analgesic, anti-inflammatory, antispasmodic, and anticonvulsant properties 79. Researchers have identified numerous pain-inducing toxin peptides from this species, including BmKI, BmP01, and ANEP. BmKI is a classic α-NaTx that prolongs the opening of sodium channels, disrupting neuronal signal transmission and causing persistent pain and muscle spasms in victims 41. BmP01, another peptide, induces pain by activating TRPV1 channels, making it the first scorpion toxin known to target TRPV1 73. Studies demonstrate that BmP01 has dose-dependent activity with strong pH-dependent effects, enhancing TRPV1 currents through a synergistic mechanism with protons. The ANEP binds to receptor site 4 on sodium channels, inhibiting normal inactivation and causing prolonged depolarization of neurons, leading to overexcitation and subsequently intense pain 21.

*Mesobuthus eupeus*, a widely distributed scorpion species known for its moderate toxicity, can still induce significant pain and local reactions despite its venom being less lethal than that of more dangerous scorpions. MeuNaTxα-1, one of the toxins isolated from this species, has been shown to induce thermal hyperalgesia in mice, making it a valuable tool for pain research 80. Other related toxins, MeuNaTxα-2 and MeuNaTxα-5 exhibit extensive sequence diversity at their carboxyl termini. Despite this variation, these peptides share conserved structural residues, suggesting that they may retain similar functions in pain induction 81, 82.

The venom of *Centruroides elegans* contains peptides such as CeII8 and CeII9, each exhibiting distinct physiological effects. CeII8 inhibits peak sodium currents and selectively targets Nav1.7 channels, prolonging the sodium channel open state by interfering with inactivation, thereby extending the duration of nerve excitation 83. CeII9, on the other hand, specifically targets Nav1.4 channels, which play a crucial role in generating and propagating action potentials in muscle fibers 83. This targeted action of CeII9 on muscle-related sodium channels may result in symptoms such as muscle paralysis or spasms, further demonstrating the specialization of scorpion toxins in targeting various ion channels for different physiological effects.

The peptide LmNaTx15 derived from *Lychas mucronatus* is demonstrated to have dual functionality, which inhibits the rapid inactivation of Nav channels and reduces their peak currents, with varying effects across different sodium channel subtypes 84. *In vivo* studies in mice have shown that LmNaTx15 induces severe nociceptive pain, confirming its role in pain modulation. Similarly, AmmVIII from *Androctonus mauretanicus mauretanicus* elicits pain-related behaviors in mice, causing rapid hypersensitivity to be mechanical and thermal stimuli 85. Additionally, AaHII, a classic α-toxin from *Androctonus australis*, enhances pain perception by modulating voltage-gated sodium channels 11. CvIV4, an α-NaTx isolated from *Centruroides vittatus*, is also known to induce pain in mammals 22.

In *Urodacus manicatus*, the venom peptide WaTx specifically targets the TRPA1 channel. WaTx activates TRPA1 by binding to intracellular sites, extending its open state and reducing calcium permeability without impacting other TRP channels 19. This selectivity underscores WaTx's unique mechanism among TRPA1 activators, potentially broadening our understanding of TRP channel modulation by venom components.

Beyond these scorpion toxin peptides, numerous others have been identified that can induce pain through distinct structural and functional mechanisms. These peptides are able to selectively target sodium channels, particularly those involved in pain transmission, such as Nav1.7 and Nav1.8. Sodium channels are essential for nerve signal conduction and are critical in generating and propagating action potentials. By altering the activation threshold of these channels or extending their open duration, scorpion toxins cause excessive neuronal depolarization, robust nerve signal transmission, and intense pain. Table 1 summarizes specific scorpion toxin peptides associated with pain induction, highlighting the diverse mechanisms by which scorpion venom impacts sodium channel function and contributes to pain. Anyway, the pain induced by scorpion venom peptides is almost considered to be a type of nociceptive pain.

## 4. Molecular mechanism of pain induced by scorpion venom peptides

The pain-inducing effects of scorpion toxic peptides are an evolutionary strategy that enables scorpions to both defend themselves and hunt effectively. Once these toxins enter the prey's body, they disrupt the nervous system, causing intense pain or paralysis, which immobilizes the prey and prevents resistance 92. This combination of pain and paralysis makes it difficult for the prey to escape, allowing the scorpion to safely capture and consume its meal. Additionally, scorpions use their venom as a potent defence mechanism, where the excruciating pain quickly deters predators or potential threats 93. The rapid onset of pain prevents predators from pursuing further, protecting the scorpion from additional harm 94. This swift and effective pain response significantly enhances the scorpion's chances of survival when confronted by natural enemies. Most scorpion toxins responsible for inducing pain are sodium channel toxins, which play a critical role in the excitation and signal transmission of neurons 95. Under normal circumstances, when neurons are stimulated, the cell membrane depolarizes, and voltage-gated sodium channels open, allowing sodium ions to flow into the cell. This influx causes the membrane potential to rise sharply, triggering an action potential essential for nerve signal transmission 96. After depolarization, sodium channels quickly inactivate while potassium channels open, allowing potassium ions to exit the cell and return the neuron to its resting state. The orderly process of opening, inactivating, and closing of sodium channels is fundamental for proper neuronal excitability and signal transduction 97. However, sodium channel toxins disrupt this critical process, leading to abnormal neuronal activity and the sensation of pain. While most pain-inducing scorpion toxins act on sodium channels, not all venom components contribute to pain. Unlike sodium channel scorpion toxins, CaTx and ClTx from scorpion venom do not directly cause pain, as their primary function is to modulate ion fluxes involved in neurotransmitter release, cell volume regulation, and other physiological processes. In some venomous animals, such as the sea snail, calcium channel inhibitors like MoVIA and MoVIB can exhibit analgesic properties by suppressing pain signal transmission 98. This contrast in toxin function highlights the diverse evolutionary strategies employed by scorpions to capture preys and defend against threats.

### 4.1. Mechanism of pain induced by α-NaTx

**Figure 3:**
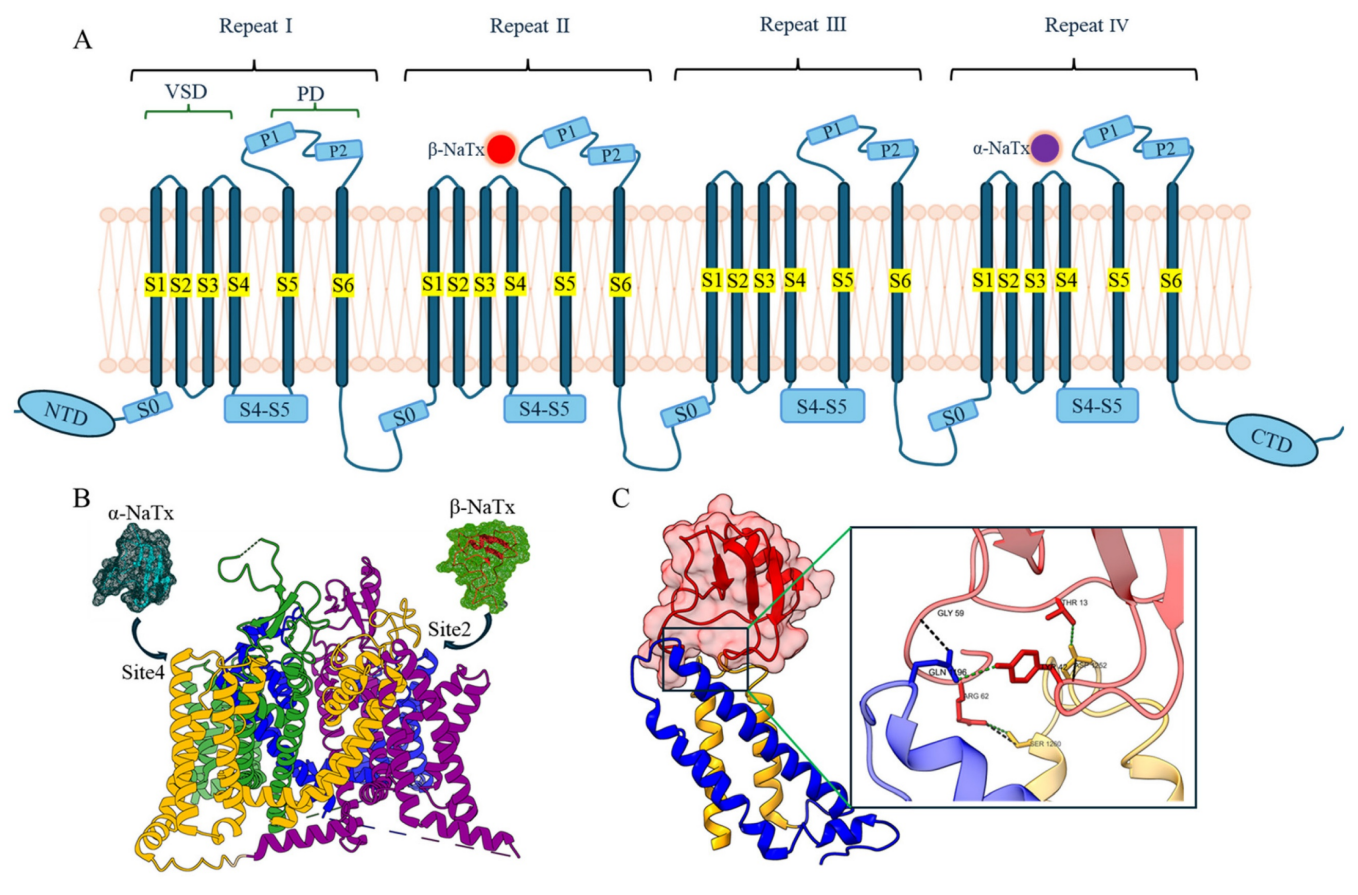
Direct interaction of the typical α-NaTx or β-NaTx and the corresponding sodium channels. (A) Topological diagram of the interaction mode between α-NaTx or β-NaTx and sodium channels. (B) Interaction map between α-NaTx or β-NaTx and sodium channels. The sodium channel human Nav1.8 is used as an example (PDB code: 7WE4), where the green part is site 1, the blue part is site 2, the purple part is site 3, and the yellow part is site 4. OD1 is used as an example of α-NaTx (PDB code: 4HHF), and Cn2 as an example of β-NaTx (PDB code: 1CN2). (C) AaHII (red) interacts with S3-S4 (green) of Nav1.7. AaHII interacts with specific amino acid residues of Nav1.7.

NaTx binds to specific sites on sodium channels, disrupting their normal inactivation process and prolonging the channels' open state. This leads to a continuous influx of sodium ions into nerve cells, resulting in a cascade of electrophysiological abnormalities that trigger excessive neuronal excitation and intense pain 99. The α-NaTx selectively binds to the S3-S4 loop of Domain IV (voltage-sensing domain IV, VSD4) in sodium channels, stabilizing the activated state of the voltage sensor to block fast inactivation and prolong channel opening. By binding to this region, α-NaTx effectively blocks the sodium channel's inactivation mechanism, preventing it from closing after depolarization 31. As a result, sodium ions flow uninterrupted into the neuron, causing a prolonged action potential. This persistent influx of sodium ions keeps the cell membrane in a depolarized state, preventing the neuron from repolarizing and returning to its resting state 100, 101. Since the sodium channels remain open for an extended period, neurons remain in a heightened state of excitation, continuously generating abnormal action potentials. These abnormal signals are transmitted to the central nervous system, where they amplify and prolong the pain sensation. The prolonged opening of sodium channels, along with the constant flow of sodium ions, lead to sustained neuronal overactivity. Even without new stimuli, the neuron remains excited, continuously firing action potentials. This results in intense pain perception, often described as tingling, burning, or persistent severe pain 102. Because α-NaTx directly interferes with the sodium channel's inactivation process, the nervous system experiences ongoing, abnormal electrical signaling, which manifests as extreme pain. The pain induced by α-NaTx is typically severe and continuous, as neurons keep firing action potentials due to the sodium channels' prolonged open state, leading to persistent and overwhelming pain. As shown in Figure 3, AaHII blocks the rapid inactivation of the sodium channel by embedding into the outer groove of VSD4, leading to sustained sodium influx 103. AaHII interacts with specific amino acid residues of VSD4 to form multiple contact points, which affect conformational changes in the channel, leading to sustained neural signalling and enhanced pain perception.

### 4.2. Mechanism of pain induced by β-NaTx

**Figure 4:**
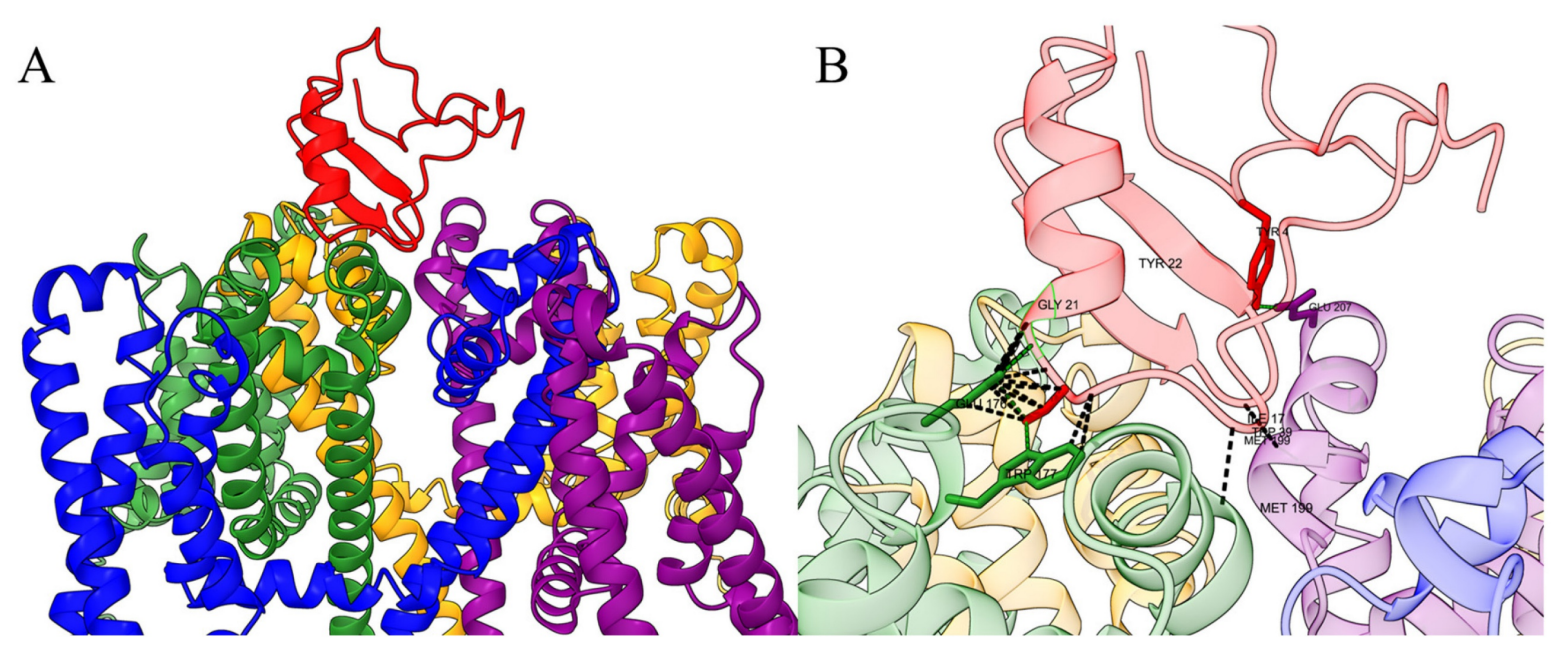
(A) Schematic diagram of the interaction between Ts1 toxin and Nav channels. The structure of Ts1 was obtained from the Protein Data Bank (PDB code: 1NPI), while the structure of the Nav channel was represented by a single domain of NavAb, a prokaryotic sodium channel from *Arcobacter butzleri* (PDB code: 4EKW). Use AlphaFold3 to predict the interaction between the Ts1 and NavAb. (B) Ts1 interacts with specific amino acid residues of NavAb.

Unlike α-NaTx, which directly prolongs the open state of sodium channels, β-NaTx alters the voltage-sensing structure of the channel, making neurons responsive to much weaker stimuli. This shift in sensitivity induces abnormal electrical signaling and severe pain 45. The β-NaTx interacts with the S3-S4 loop of Domain II (voltage-sensing domain II, VSD2), shifting the voltage-dependent activation threshold to hyperpolarized potentials, thereby enabling subthreshold neuronal excitation. As a result, sodium channels open even before the neuron reaches its normal activation threshold, generating action potentials and disrupting normal nerve signal transmission 104. Under typical conditions, sodium channels only activate and generate action potentials when neurons are exposed to sufficiently strong stimuli. However, when β-NaTx acts on sodium channels, it significantly lowers the activation threshold, causing sodium channels to open with minimal or even no stimulation. This leads to more frequent sodium ion entry into nerve cells, triggering repeated membrane potential depolarisation and causing a nerve signal transmission surge 33, 105. Unlike α-NaTx, β-NaTx does not directly interfere with the inactivation process of sodium channels. However, because it continuously triggers new depolarization events, neurons fire action potentials repeatedly, resulting in intense and persistent pain. This mechanism highlights how β-NaTx creates heightened neuronal sensitivity, leading to the overexcitation of pain pathways even in response to weak or absent stimuli, ultimately amplifying the sensation of pain. The mode of action of β-NaTx on sodium ion channels is shown in Figure 4 76.

### 4.3. Mechanism of pain induced by TRPTx

While not many scorpion toxins are known to act on TRP channels, those identified offer valuable insights into how these toxins elicit pain in their victims. BmP01 binds to and activates the TRPV1 channel, causing it to open and allowing cations, particularly calcium ions, to flow into the neuron 73. This influx leads to the depolarization of the neuronal membrane, triggering action potentials that are transmitted to the brain and interpreted as pain. In contrast to many other toxins, WaTx possesses the unique ability to penetrate the cell membrane without the need for specific receptors or channels 19. This enables WaTx to access the intracellular domain of TRPA1 and directly modulate its activity from within the cell. By binding to the intracellular region of TRPA1, WaTx triggers its activation, resulting in the depolarization of the neuronal membrane. Like BmP01's effect on TRPV1, this depolarization generates an action potential that is relayed to the central nervous system as a pain signal. Toxins from other venomous animals can enhance our understanding of how TRP channels mediate pain. For example, the spider toxin VaTx can activate TRPV1 channels and induce pain 106. In addition to generating action potentials through depolarization, this process is accompanied by the release of pro-inflammatory molecules, which further increase the sensitivity of sensory neurons. This amplification of the pain response leads to inflammatory pain, characterized by redness, swelling, and hypersensitivity. The centipede toxin RhTx is a potent pain-inducing peptide that specifically targets the heat activation mechanism of the channel TRPV1 107. By interacting with TRPV1, RhTx effectively lowers the temperature threshold required for channel activation, causing TRPV1 to open at temperatures below normal. This results in the triggering of heat pain signals even in response to mild or non-painful heat conditions. These toxin peptides from other venomous animals can help us understand how scorpion toxins induce pain through TRP channels. As research into scorpion toxin peptides continues, more peptides capable of activating TRP channels will likely be discovered.

## 5. Conclusions

Scorpion venom peptides are an effective biochemical weapon in nature that can quickly cause pain and paralysis, helping scorpions hunt and defend themselves. The mechanism of inducing pain is mainly through affecting the function of sodium and calcium channels, leading to excessive excitation of neurons and persistent pain. This review mainly introduced the structure and function of a variety of scorpion toxin peptides, emphasizing their effects on the nervous system and their important role in pain transmission. Future research is expected to use the properties of these venom peptides to develop new treatments for pain-related diseases and further reveal the functions of ion channels in physiological and pathological states. A deeper understanding of the mechanism of action of these toxins can provide a theoretical basis for designing new drugs, thereby improving pain management and treatment.

## Figures and Tables

**Figure 1:**
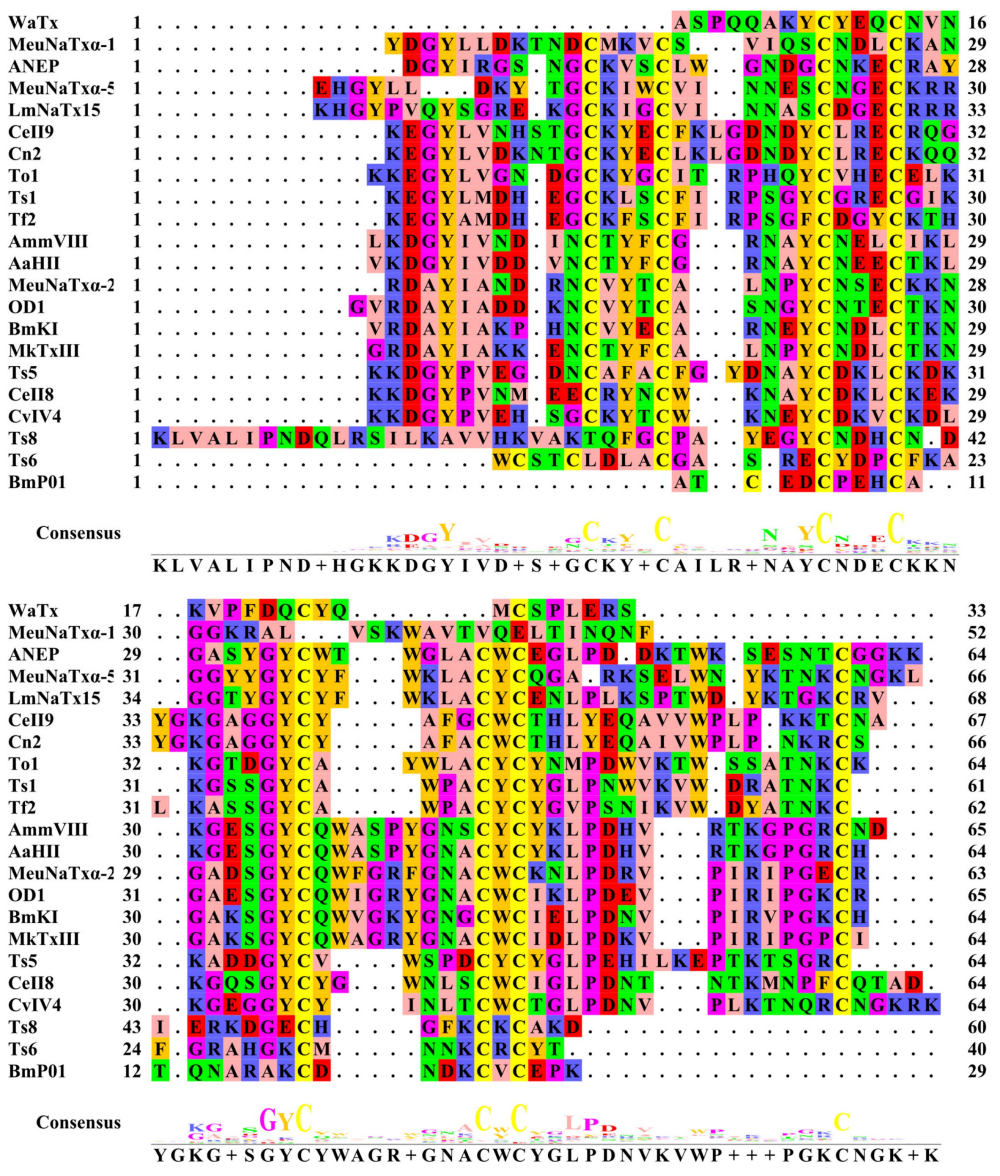
Multiple sequence alignment of amino acid sequences of pain-causing scorpion venom peptides. Multiple sequence alignment of amino acid sequences was produced on the EMBL-EBI website (https://www.ebi.ac.uk/jdispatcher/msa/clustalo). Zappo was used to color amino acids.

**Figure 2:**
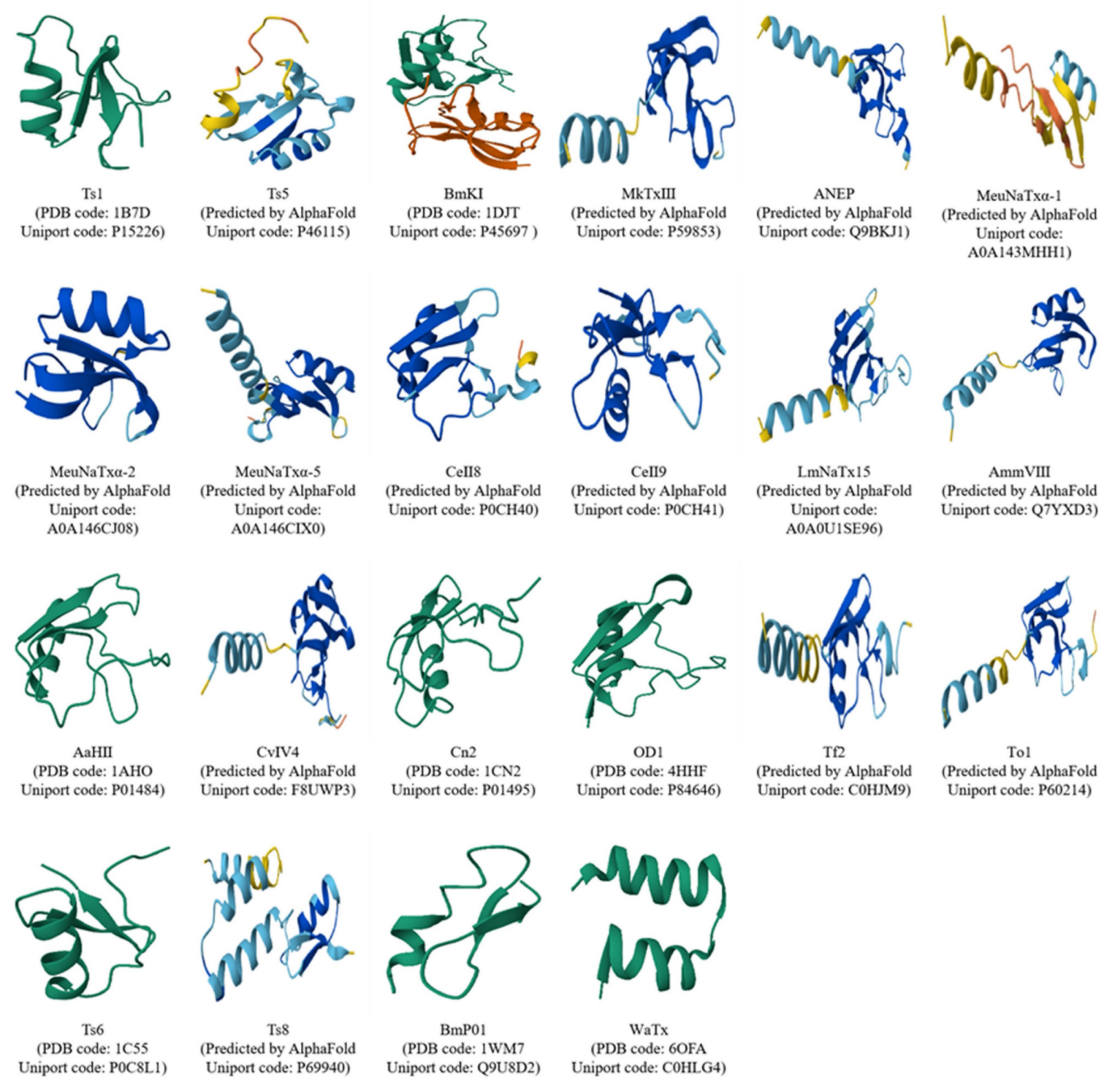
High-level structure diversity of pain-causing scorpion venom peptides. The structures of some undetermined scorpion venom peptides were predicted by AlphaFlod3 91.

**Table 1:** Scorpion venom peptides with pain-inducing properties.

Toxins	Uniport Codes	Scorpion species	Targets	pI	Length (aa)	References
Ts1	P15226	*Tityus serrulatus*	Almost all Nav	8.668	61	42
Ts5	P46115	*Tityus serrulatus*	Almost all Nav	5.143	64	77
BmKI	P45697	*Buthus martensii* Karsch	Nav1.7	8.17	64	41
MkTxIII	P59853	*Buthus martensii* Karsch	Nav1.7	8.463	64	86
ANEP	Q9BKJ1	*Buthus martensii* Karsch	Nav1.7	7.673	64	21
MeuNaTxα-1	A0A143MHH1	*Mesobuthus eupeus*	Nav1.2, Nav1.3 and Nav1.7	7.849	52	80
MeuNaTxα-2	A0A146CJ08	*Mesobuthus eupeus*	Nav1.4	8.492	63	81
MeuNaTxα-5	A0A146CIX0	*Mesobuthus eupeus*	Nav1.3, Nav1.4, Nav1.5, Nav1.6 and Nav1.7	8.946	66	82
CeII8	P0CH40	*Centruroides elegans*	Nav1.7	8.156	64	83
CeII9	P0CH41	*Centruroides elegans*	Nav1.4	8.156	67	83
LmNaTx15	A0A0U1SE96	*Lychas mucronatus*	Nav1.2, Nav1.3, Nav1.4, Nav1.5 and Nav1.7	9.107	68	84
AmmVIII	Q7YXD3	*Androctonus mauretanicus*	Almost all Nav	8.156	65	85
AaHII	P01484	*Androctonus australis*	Nav1.2 and Nav1.7	7.673	64	11
CvIV4	F8UWP3	*Centruroides vittatus*	Nav1.2, Nav1.3, Nav1.4, Nav1.7 and Nav1.8	8.814	64	22
Cn2	P01495	*Centruroides noxius*	Nav1.6	8.156	66	87
OD1	P84646	*Odonthobuthus doriae*	Nav1.7, Nav1.3, Nav1.4 and Nav1.6	7.673	65	88
Tf2	C0HJM9	*Tityus fasciolatus*	Nav1.3	8.17	62	89
To1	P60214	*Tityus obscurus*	Nav1.3 and Nav1.6	8.434	64	20
Ts6	P0C8L1	*Tityus serrulatus*	Kv1.2 and Kv1.3	8.507	40	78
Ts8	P69940	*Tityus serrulatus*	Kv4.2	8.287	60	90
BmP01	Q9U8D2	*Buthus martensii* Karsch	TRPV1	4.952	29	73
WaTx	C0HLG4	*Urodacus manicatus*	TRPA1	6.186	33	19
